# Genetic Diagnosis Spectrum and Multigenic Burden of Exome-Level Rare Variants in a Childhood Epilepsy Cohort

**DOI:** 10.3389/fgene.2021.782419

**Published:** 2021-12-21

**Authors:** Ruen Yao, Yunqing Zhou, Jie Tang, Niu Li, Tingting Yu, Yingzhong He, Cuijin Wang, Jiwen Wang, Jian Wang

**Affiliations:** ^1^ Department of Medical Genetics and Molecular Diagnostic Laboratory, Shanghai Children’s Medical Center, School of Medicine, Shanghai Jiao Tong University, Shanghai, China; ^2^ Department of Neurology, Shanghai Children’s Medical Center, School of Medicine, Shanghai Jiao Tong University, Shanghai, China

**Keywords:** epilepsy, genetic diagnosis, polygenic risk, ion channel, synaptic

## Abstract

Childhood epilepsy is a considerably heterogeneous neurological condition with a high worldwide incidence. Genetic diagnosis of childhood epilepsy provides the most accurate pathogenetic evidence; however, a large proportion of highly suspected cases remain undiagnosed. Accumulation of rare variants at the exome level as a multigenic burden contributing to childhood epilepsy should be further evaluated. In this retrospective analysis, exome-level sequencing was used to depict the mutation spectra of 294 childhood epilepsy patients from Shanghai Children’s Medical Center, Department of Neurology. Furthermore, variant information from exome sequencing data was analyzed apart from monogenic diagnostic purposes to elucidate the possible multigenic burden of rare variants related to epilepsy pathogenesis. Exome sequencing reached a diagnostic rate of 30.61% and identified six genes not currently listed in the epilepsy-associated gene list. A multigenic burden study revealed a three-fold possibility that deleterious missense mutations in ion channel and synaptic genes in the undiagnosed cohort may contribute to the genetic risk of childhood epilepsy, whereas variants in the gene categories of cell growth, metabolic, and regulatory function showed no significant difference. Our study provides a comprehensive overview of the genetic diagnosis of a Chinese childhood epilepsy cohort and provides novel insights into the genetic background of these patients. Harmful missense mutations in genes related to ion channels and synapses are most likely to produce a multigenic burden in childhood epilepsy.

## Introduction

Childhood epilepsy is an exceptionally heterogeneous neurological condition with a high incidence of 5–8 in 1,000 in China (Trinka et al., 2019). Genetic diagnosis of childhood epilepsy provides the most accurate pathogenetic evidence and is crucial for providing disease-specific treatments. Monogenic forms of seizure disorders tend to manifest earlier in life, presenting as childhood epilepsy, with a broad clinical spectrum, ranging from benign, self-limited epilepsies to severe epileptic encephalopathies (Sands and Choi, 2017; Jiang et al., 2021). Hundreds of genes have been associated with epilepsy, and the list is increasing continuously (Wang et al., 2017). With the aforementioned genes, diagnosis of epilepsy based on clinical features and biochemical investigations is now moving to the genetic testing stage. Clinical application of next-generation sequencing (NGS) targeting these genes has contributed greatly to the identification of causative genetic mutations (Lindy et al., 2018; Hebbar and Mefford, 2020). Diagnostic application of NGS for cohorts of adult and children with epilepsy have gradually evolved from chromosomal microarray analysis (CMA) to target panel sequencing and subsequently expanded exome range, and the diagnostic yield ranged from 23 to 42% (Yang et al., 2019; Johannesen et al., 2020). Retrospective analysis of clinical and genetic diagnoses of childhood epilepsy in different cohorts and ethnic groups could lead to a better understanding of the genetic etiology of epilepsy and provide information on prognosis, treatment, and genetic counseling in the era of precision medicine.

Studies have revealed that genetic factors similarly play a regulatory role in acquired epilepsies resulting from trauma, stroke, neoplasm, infection, or congenital malformations (Thomas and Berkovic, 2014). Moreover, abundant genomic data of epilepsy patients have enabled research into common underlying genetic risks, and polygenic burden has been proven to be significantly enriched in multiple cohorts of patients with epilepsy (Leu et al., 2019; Moreau et al., 2020). This polygenic background information is not diagnostic; however, it suggests the possibility of rare exome variants and their combinations in epilepsy pathogenesis (Lindy et al., 2018). The contribution of rare exome-level variants that do not fit the criteria for pathogenetic diagnosis under the American College of Medical Genetics and Genomics/Association for Molecular Pathology (ACMG/AMP) guidelines for Mendelian genetic disease still deserves exploration and study. In this study, we retrospectively analyzed the clinical and genetic characteristics of Chinese childhood epilepsy patients and examined possible exome-level rare variant accumulation as a polygenic risk factor for childhood epilepsy.

## Materials and Methods

### Study Cohort

In total, 294 childhood epilepsy patients from Shanghai Children’s Medical Center, Department of Neurology, were recruited in this study. All recruited patients have been confirmed to have no known etiology and met the following criteria for epilepsy established by the International League Against Epilepsy (ILAE): 1) at least two unprovoked (or reflex) seizures occurring >24 h apart, 2) one unprovoked (or reflex) seizure and a ≥60% chance of recurrent seizures over the next 10 years, or 3) diagnosis of an epilepsy syndrome (Moreau et al., 2020). The epileptic syndrome was identified by clinical manifestations and electroencephalogram (EEG) features according to the classification and terminology of the ILAE Commission (Fisher et al., 2017). The Ethics Committee at Shanghai Children’s Medical Center approved the study. Written informed consent was obtained from the parents of all participants.

### Clinical Genetic Diagnosis of Childhood Epilepsy

Peripheral blood samples were collected from patients and their parents. The SureSelect Human All Exon V6 enrichment kit (Agilent, Santa Clara, CA, United States) and ClearSeq Inherited Disease Panel enrichment kit (Agilent) was used to prepare the sequencing library. The experimental process included enzyme digestion of DNA fragments, library hybridization, and capture library amplification and purification. The Hiseq2500 sequencing platform (Illumina, Inc., San Diego, CA, United States) was used for high-throughput sequencing. Fastqc (Babraham Research Institute, Cambridge, UK) and Fastp (Visible Genetics, Inc., Toronto, Canada) were used for data quality control and the removal of the adaptor sequence. Speedseq (Ira Hall Lab, St. Louis, MO, United States) was aligned to the reference genome (Chiang et al., 2015), and bamdst and mosdepth were used to count the sequencing indexes of BAM files after alignment, including the mapping rate, PCR (polymerase chain reaction) duplication rate, average sequencing depth, and coverage rate. The Genome Analysis Toolkit (Broad Institute, Cambridge, MA, United States) was used to detect the variations in the BAM file that passed the quality control, and a VCF format file was generated. Variations in VCF files were annotated using Ingenuity Variant Analysis (IVA) (Ingenuity Systems, Redwood City, CA, United States) and Translational Genomics Expert (TGex) platforms. Finally, Sanger sequencing was used to verify the candidate gene mutation, and the genotypes of the family members were determined. All suspected variants were confirmed by Sanger sequencing and validated using parental test results. Copy number variants (CNVs) were identified using open-source CNVkit software, which is a tool kit that can infer and visualize copy numbers from targeted DNA sequencing data (Talevich et al., 2016). Previously aligned exome data (BAM files) for sequencing variant screening were used again as inputs. Normal references for CNV identification were constructed based on sequencing data generated under the same protocol and experimental conditions from 10 normal males and 10 normal females with no pathogenic CNVs validated by chromosomal microarray analysis. Individual CNVs were identified using default CNVkit settings.

Clinical information and genetic characteristic analysis of childhood epilepsy cohort.

Detailed clinical information included age, onset age, seizure onset type, brain magnetic resonance imaging (MRI) findings, electroencephalogram results, prognosis, family history, and other related clinical phenotypes.

Genetic variants detected in the childhood epilepsy cohort were manually classified according to the method recommended by the American College of Medical Genetics and Genomics. The genetic diagnostic rates of the cohort were calculated according to the clinical information within different groups.

### Exome-Level Polygenic Risk Analysis for Childhood Epilepsy Cohort

The possible exome-level polygenic risks for childhood epilepsy were evaluated based on exome data from 157 childhood epilepsy patients (46 with a diagnosed monogenic disorder) in our study. In total, 310 normal control exome data and 210 exome sequencing data of patients diagnosed with aplastic anemia and with any confirmed seizure history were used as references. Variants were filtered first by quality scores recalibrated using VQSR at the recommended 99% sensitivity tranche and subsequently filtered with a high batch frequency ( >5%). Genes with known functions of metabolism, ion channels, cell growth, and regulatory and synaptic functions were selected according to GeneCards Inferred Functionality Scores (GIFtS) and relevance scores for variant distribution analysis (Supplementary Table S1). The number of likely gene-disruptive variants (LGD), including loss-of-function-related variants in genes with constraint score pLI ≥0.9, the number of missense variants with CADD score >30 in genes with missense z-score >3.09 (MIS30), and their distributions among previously selected gene lists with various functions, were compared between groups. Exome-level polygenic risk enrichment frequency was calculated based on the occurrence of LGD and MIS30 variants in separate gene lists compared with the number of rare variants in the same lists. Variants confirmed as monogenic disorder diagnoses were excluded when counting the number of variants contributing to polygenic risk scores.

## Results

### Demographic Data of Childhood Epilepsy Cohort

In total, 294 individuals diagnosed based on the ILAE criteria for epilepsy who underwent clinical exome panel or whole-exome sequencing were recruited in this study. The average age of the cohort was 5 years 7 months, with a median age of 6 years 5 months. Overall, 245 patients had a first documented seizure from the neonatal period to the age of 13 years, with an average onset age of 2 years 9 months and median onset age of 1 year 6 months. From the available clinical information, 218 patients had EEG results, and 220 patients had brain MRI results. EEG results were abnormal in 141 cases, including diffuse epileptiform discharge, focal seizure, and myoclonic seizure, accounting for approximately 64.67% of the participants. Brain MRI results were abnormal in 99 cases, including white matter lesions, mild atrophy, and pachygyria, and 43 patients were clinically identified as having epilepsy syndrome, including Dravet syndrome, West syndrome/LGS syndrome/Ohtahara syndrome, and BECT/BFNS. Seizures were focal-onset in 133 patients and generalized-onset in 81. Varying levels of developmental delay or intellectual disability were present in 95 patients (Table 1). The percentage of patients with presumed genetic epilepsy with no associated comorbidities was 67.69% (199/294). Anti-seizure medication and prognosis information were available for 186 patients, among whom 99 were seizure-free after treatment, 20 had 75% seizure reduction, 17 had 50% seizure reduction, and 50 received invalid treatment. Detailed clinical information of the cohort is presented in Supplementary Table S2.

**TABLE 1:** Demographic data of the childhood epilepsy cohort.

Characteristics	Entire cohort
Age	Average: 5y7m Median:6y5m
Gender (male/female)	180/114
Seizure onset age	Average: 2y9m Median:1y6m
Seizuer onset type	
Generalized onset	90
Focal onset	124
N/A	80
Epilepsy syndrome	
Dravet Syndrome	9
West Syndrome/LGS syndrome/Ohtahara syndrome	21
BECT/BFNS	9
Abnormal ECG	141
Abnormal brain MRI	112
Family history of epilepsy	57
Developmental delay	51

### Genetic Diagnosis of Epilepsy Cohort and Geno-Phenotype Correlations

**FIGURE 1:**
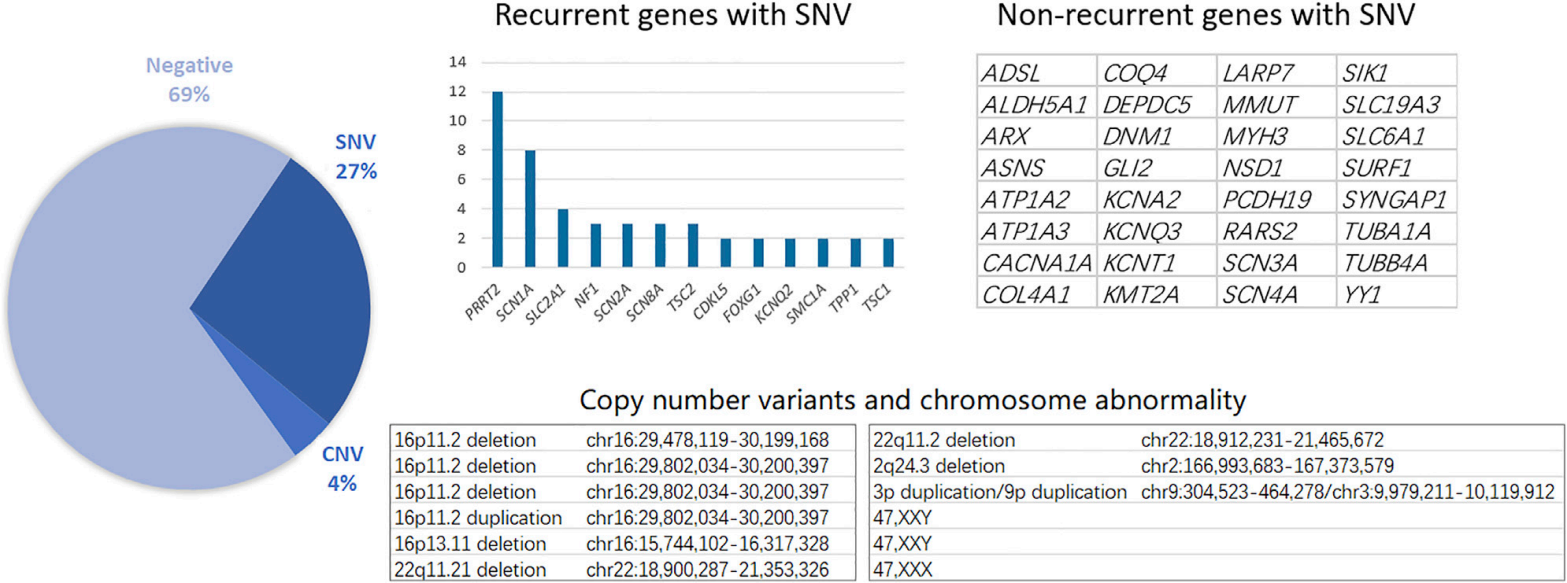
Genetic diagnosis spectrum and pathogenic SNV and CNV in the childhood epilepsy cohort.

Genetic testing, including single nucleotide variants (SNVs) and CNVs, reached a total diagnostic rate of 30.61%, revealing the molecular pathogenesis in 90 patients: 12 with CNVs and 78 with SNVs. The most affected chromosome was chr16, with three 16p11.2 deletions, one 16p11.2 duplication, and one 16p11.31 duplication. Among the 78 patients identified with epilepsy-related SNVs, the most frequently affected genes were *SCN1A* and *PRRT2*. Similarly, recurrent SNV was detected in other genes, including *SLC2A1*, *NF1*, and *SCN2A* (Figure 1). Other non-recurrent SNVs were identified in 32 different genes. According to the gene categories suggested in a previous study (Sands and Choi, 2017), 43 genes with SNVs detected in our study were epilepsy genes, seven were neurodevelopmental-associated genes, 21 were epilepsy-related genes, and two were genes putatively associated with epilepsy. Six genes with SNVs were not listed in the previous gene list, including *ALDH5A1*, *MMUT*, *RARS2*, *SURF1*, *LARP7*, and *YY1* (Table 2).

**TABLE 2:** Neurological involement information of six genes detected outside the exsiting epilepsy-associated gene list.

Gene	Disease	Inheritance	Neurological involement	Reference
*ALDH5A1*	Succinic semialdehyde dehydrogenase deficiency	AR	Hypotonia and developmental delay, nonprogressive ataxia, hyporeflexia, behavioral dysregulation, obsessive-compulsive disorder, anxiety disorder, and ADHD. **29.17% (7/24) patients have seizure.**	Neurology. 2020; 95 (19):e2675-e2682.
*MMUT*	Methylmalonic aciduria	AR	Seizure, psychomotor retardation, poor feeding, respiratory distress, loss of consciousness, and muscle tone abnormality. **30.77% (16/52) patients have seizure.**	Pediatr Radiol. 2008; 38 (10):1054-61.
*RARS2*	Pontocerebellar hypoplasia		Encephalopathy with intractable seizures and severe developmental delay.	J Med Genet. 2021 Mar 5; jmedgenet-2020-107497.
*SURF1*	Mitochondrial complex IV deficiency nuclear type 1/Charcot-Marie-Tooth disease, type 4K	AR	Hypotonia, oculomotor abnormalities, ataxia, tremor, and brisk tendon reflexes. **One patients reported with seizure**	Eur J Pediatr. 1994; 153 (2):133-5.
*LARP7*	Alazami syndrome	AR	Intellectual disability, delayed psychomotor development, unstable gait. **One (1/5) patient has been reported with seizure.**	Eur J Med Genet. 2019; 62 (3):161-166.
*YY1*	Gabriele-de Vries syndrome	AD	Delayed psychomotor development, variable cognitive impairment, speech delay, and behavioral problems, such as anxiety and autistic features. **One (1/10) patient has been reported with seizure.**	Am J Hum Genet. 2017; 100 (6):907-925.

**FIGURE 2:**
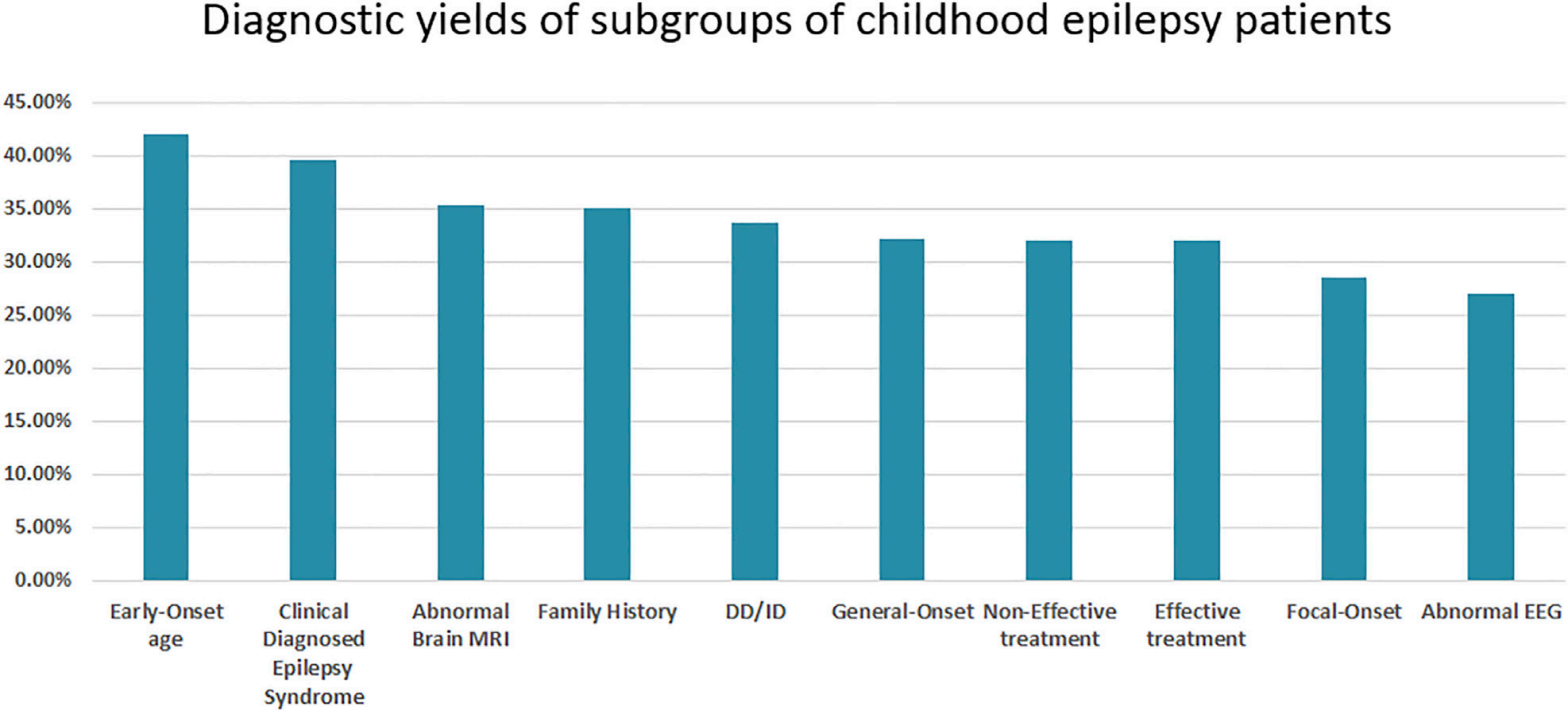
The diagnostic yield of the subgroups of childhood epilepsy patients.

The diagnostic rates for different subgroups based on treatment effect, seizure onset age and type, and other detailed clinical information were calculated. Further, patients with early-age onset and clinically diagnosed epilepsy syndromes had higher genetic diagnostic rates of 42.06 and 39.53%, respectively. Patients with abnormal EEG results and focal onset had lower genetic diagnostic rates of 28.57 and 26.95%, respectively. Interestingly, patients with a family history had a genetic diagnostic rate of only 35.09% (Figure 2).

Enrichment frequency of LGD/MIS30 variants and exome-level polygenic risks for childhood epilepsy.

**FIGURE 3:**
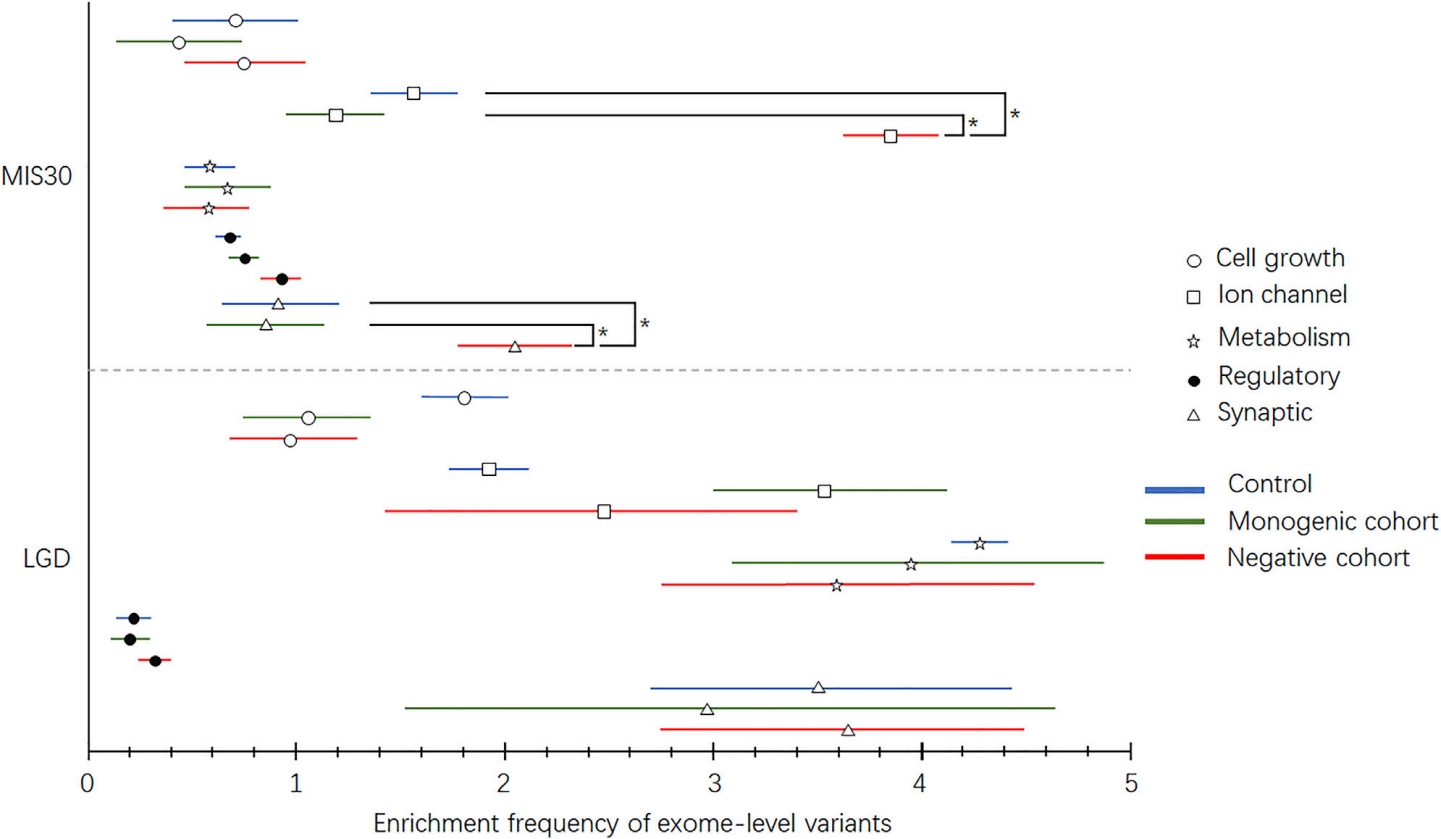
Enrichment frequency of exome MIS30 and LGD variants in different functional categories. MIS30 variants’ enrichment in patients with a negative monogenic diagnosis is significantly higher than that in the control and monogenic groups.

Five functional categories of the epilepsy-associated gene list were selected using keywords, such as ion channel, synaptic, metabolic, cell growth, and regulatory. Protein-coding genes with GIFtS >30 and relevance scores >4 according to the GeneCards database were included. Detailed gene lists used for subsequent analysis and their parameters are listed in Supplemental Table S1. Exome-level polygenic risks were evaluated by the enrichment frequency difference of rare LGD/MIS30 variants in the five functional categories of epilepsy-associated genes between patients in the monogenic diagnosis, negative genetic diagnosis, and control groups. Compared with the reference group and the monogenic diagnosis group, the undiagnosed childhood epilepsy group bore two to three times more MIS30 variants in ion channel- and synaptic function-related genes (Figure 3, Supplemental Table S4). LGD and MIS30 variant distribution in the gene categories of cell growth and proliferation, metabolic, and regulatory function showed no significant difference.

## Discussion

The major underlying etiology of epilepsy is genetic, especially in early-onset childhood epilepsy; thus, genetic testing has been widely accepted and applied in the clinical diagnosis of epilepsy (Costain et al., 2019; Rochtus et al., 2020). Although multiple options are available for the diagnostic investigation of the complex genetic architecture of epilepsy, high-throughput sequencing is currently accepted as the first-tier choice (Dunn et al., 2020). Targeted panel sequencing offers superior cost performance and reliable diagnosis, while whole-exome sequencing and whole-genome sequencing further increase the diagnostic yield for novel gene discovery. The advantages of high-throughput sequencing include deletion/duplication analysis, thus gradually removing chromosomal microarray analysis from clinical applications. The diagnostic yield of high-throughput sequencing in our childhood epilepsy cohort was 30.61% (90/294), including CNVs and SNVs. The mutation spectrum of our cohort consisted of 12 CNVs and SNVs in 45 genes. CNVs affecting the 16p11.2 genetic locus are associated with a range of neurodevelopmental disorders, including autism spectrum disorder, intellectual disability, and epilepsy, and were detected in three patients in our cohort (Rein and Yan, 2020). Animal models of 16p11.2 deletions or duplications recapitulate many core behavioral phenotypes, including social and cognitive deficits, and exhibit altered synaptic function across various brain areas. Therefore, treatment of cognitive deficits in these children should be emphasized when epilepsy is controlled.

Ion channel gene variants accounted for the majority of monogenic diagnoses for childhood epilepsy in our study. We categorized the monogenic diagnoses into the four classifications of epilepsy-associated genes proposed by Wang et al. (Wang et al., 2017). Pure epilepsy symptoms manifested in 43 patients with epilepsy genes. Seven patients had neurodevelopmental-associated genes. Twenty-one patients had epilepsy-related genes, and two had putatively associated epilepsy genes. Six patients were identified with variants in genes not currently categorized into the four classifications. Further investigation of these six cases revealed three possible explanations (Table 2). Patients with metabolic disorder-related genes (*ALDH5A1* and *MMUT*) are known to develop neurological disorders; however, seizures are not always the first or the most remarkable symptom requiring medication. Two patients were diagnosed with genes associated with mitochondria-related disorders (*RARS2* and *SURF1*). Mitochondrial diseases usually affect multiple organs and exhibit strong clinical heterogeneity (Rahman, 2019). Some patients may only have nervous system abnormalities or epilepsy as the main manifestation; however, patients previously reported with epilepsy bearing variants in *RARS2* and *SURF1* are rare. The last two genes were newly discovered neurodevelopmental syndrome genes (*LARP7* and *YY1*), which require more cases to summarize clinical characteristics and to update the epilepsy-associated gene lists.

The genetic diagnostic yield in pediatric patients with epilepsy with different clinical features was further analyzed in our study. Patients with generalized onset had a higher diagnostic yield than patients with focal onset, which is consistent with previous research (Torabian et al., 2019). Diagnostic yield in the remaining subgroups based on clinical features was consistent with the current understanding of the genetic basis of epilepsy, as patients with early-onset seizures, clinically recognizable epilepsy syndromes, and abnormal brain MRI have a higher diagnostic yield. The diagnostic rate between patients in the effective and non-effective treatment groups did not differ significantly.

Anti-seizure medication administration usually starts immediately after the diagnosis of epilepsy; however, the turn-around time of gene testing is as long as 6–8 weeks in developing countries, such as China. Treatment targeting the pathogenesis of known epilepsy genes should improve the effectiveness of treatment, as the pathogenesis and relationship between genotypes and phenotypes are relatively clear for some epilepsy genes, especially the common ion channel genes (Møller et al., 2019). Patients with *PRRT2* mutations responded to oxcarbazepine or levetiracetam. Ketogenic diet was very effective for patients, with the *SLC2A1* variant, who were diagnosed with glucose transporter 1 deficiency. Most other patients with gene mutations, such as *SCN1A*, *KCNT1*, *CDKL5*, are more likely to be drug-resistant. Thus, adjusting anti-seizure medications according to the results of genetic testing will be the key to improving the diagnosis and treatment of epilepsy.

With the decrease in sequencing cost and the increased clinical relevance of genetics, genomic data are currently being adopted in the daily clinical practice of neurology. Clinicians should interpret and apply genetic data for the management of patients with epilepsy. The risk of generalized epilepsy in first-degree relatives of patients (siblings and children) is increased five- to ten-fold relative to the background population (Peljto et al., 2014). However, in our study of childhood epileptic patients with a family history, only 31.58% of those were monogenically diagnosed. A large proportion of the undiagnosed epilepsy population may still suffer from a genetic etiology that has not been fully revealed, leading to the assumption of a polygenic etiology.

Common variant risk has been proven to be significantly enriched in multiple cohorts of patients with epilepsy compared to controls using polygenic risk scores (Johannesen et al., 2020). Detection of common variants has a limited contribution to genetic diagnosis; however, studies have equally illustrated the possibility of measuring genetic risk by accumulating singleton loss-of-function variants in neurodevelopmental disorders (Chen et al., 2018). Thus, a comparison of the enrichment of rare but deleterious exonic variants between patients and control cohorts could provide insight into the multigenic burden of epilepsy pathogenesis.

Epilepsy-associated genes can be grouped into the following five broad functional categories: ion transport, cell growth and differentiation, regulation of synaptic processes, transport and metabolism of small molecules within and between cells, and regulation of gene transcription and translation (Symonds and McTague, 2020). Thus, we calculated the enrichment of rare but deleterious exonic variants (LGD and MIS30 variants) in the gene lists of patients with a monogenic diagnosis, those with negative results, and control cohorts for evaluation purposes. LGD variants were not significantly different between the groups in any of the five gene categories. These LGD variants are usually highly emphasized in the analysis of monogenic traits. When excluded from the WES analysis workflow, the LGD variants were less likely to be disease-related. A three-fold possibility of MIS30 variant occurrence in ion channel and synaptic genes in the undiagnosed group was identified, which may contribute to the multigenic risk of childhood epilepsy. In the process of analyzing the WES data of epilepsy patients, these MIS30 variants (most of them inherited from parents) are always classified as variants with unknown clinical significance, which cannot be used for monogenic diagnosis and cannot completely exclude the pathogenicity of the variant. Our findings suggest that the enrichment and accumulation of MIS30 variants in ion channel- and synaptic function-related genes may represent a multigenic burden affecting the severity of epilepsy or risk of epilepsy.

In conclusion, genetic testing based on exome sequencing has a satisfactory diagnostic rate in childhood epilepsy patients. However, multiple deleterious variants at the exome level supposedly contribute to the pathogenesis of epilepsy in patients without a monogenic diagnosis. Harmful missense mutations in genes related to ion channels and synapses are most likely to produce a multigenic burden in childhood epilepsy. Mutation and gene spectra associated with epilepsy should be updated frequently to improve genetic diagnosis in clinical practice. Nevertheless, further studies in large epilepsy cohorts should reveal more reliable evidence of multigenic causes of epilepsy for use in clinical diagnosis.

## Data Availability

The variants data presented in the study are deposited in the Genome Variation Map repository from the National Genomics Data Center (CNCB-NGDC), accession number GVM000284.
